# Brain structure across the lifespan: the influence of stress and mood

**DOI:** 10.3389/fnagi.2014.00330

**Published:** 2014-11-24

**Authors:** José M. Soares, Paulo Marques, Ricardo Magalhães, Nadine C. Santos, Nuno Sousa

**Affiliations:** ^1^Life and Health Sciences Research Institute, School of Health Sciences, University of MinhoBraga, Portugal; ^2^ICVS/3B’s – PT Government Associate LaboratoryBraga/Guimarães, Portugal; ^3^Clinical Academic Center – BragaBraga, Portugal

**Keywords:** aging, stress, mood, volumetry, gray matter, white matter

## Abstract

Normal brain aging is an inevitable and heterogeneous process characterized by a selective pattern of structural changes. Such heterogeneity arises as a consequence of cumulative effects over the lifespan, including stress and mood effects, which drive different micro- and macro-structural alterations in the brain. Investigating these differences in healthy age-related changes is a major challenge for the comprehension of the cognitive status. Herein we addressed the impact of normal aging, stress, mood, and their interplay in the brain gray and white matter (WM) structure. We showed the critical impact of age in the WM volume and how stress and mood influence brain volumetry across the lifespan. Moreover, we found a more profound effect of the interaction of aging/stress/mood on structures located in the left hemisphere. These findings help to clarify some divergent results associated with the aging decline and to enlighten the association between abnormal volumetric alterations and several states that may lead to psychiatric disorders.

## INTRODUCTION

Normal brain aging is an inevitable, complex, and heterogeneous process, characterized by a selective pattern of structural and functional changes. With age, the whole brain itself and many of its specific structures present volumetric alterations, mostly reductions, white matter (WM) becomes less dense and loses integrity (Walhovd et al., 2011; Fjell et al., 2013). Specifically, “normal” brain aging has been consistently characterized by a noted overall atrophy associated with a decrease in brain volume and expansion of the cerebrospinal fluid (CSF) spaces (Tamnes et al., 2013). Although total brain volume is more correlated with age after 60 years old, gray matter (GM) volume decline may begin earlier and progress gradually, frequently associated with neuronal cell death, whereas WM may start later and progress more abruptly accompanying the myelin sheath deteriorating after the fourth decade of life (Dennis and Cabeza, 2008; Gunning-Dixon et al., 2009; Lemaitre et al., 2012; Fjell et al., 2013; Tamnes et al., 2013). Specifically, both GM and WM volumetric reductions seem to be greater in the cortex than in subcortical structures, associated to a greater spatial extent in WM, with the highest effects in GM frontal lobe and in the WM superior and medial frontal and anterior cingulate regions (Raz et al., 2005; Dennis and Cabeza, 2008; Gunning-Dixon et al., 2009; Salat et al., 2009b; Voineskos et al., 2010; Sala et al., 2012).

Notably, not only do such changes occur even in highly cognitive functioning individuals (Tamnes et al., 2013; Meunier et al., 2014), but during healthy aging, many domains become also less efficient, and the brain tends to respond to all these neural changes by engaging in continuous reorganizations to keep its homeostatic control and support cognitive functions, the so-termed “brain plasticity” (Park and Reuter-Lorenz, 2009; Lovden et al., 2013). Aging quality varies according to space (brain region), time (lifespan phase), subject (individual parameters), and external influences. Understanding and characterizing the structural brain changes across the lifespan using magnetic resonance imaging (MRI), taking into account the complex combination of distinct life experience, amongst which the exposure to stressful experience and variations in mood are major factors, became one of the most prominent challenges in the comprehension of the cognitive function in middle/late ages.

Despite the limited information on the stress and mood-induced structural alterations, most studies point to reduced volumes in stressed participants in the anterior cingulate cortex, hippocampus, and amygdala (van der Werff et al., 2013; Lucassen et al., 2014). Smaller prefrontal and anterior cingulate cortex volumes have also been observed in patients with major depressive disorder (Frodl et al., 2008) and in participants with prolonged stress (Blix et al., 2013; De Brito et al., 2013). Reduced volumes in the hippocampus, caudate, and putamen have also been reported in depressed subjects (Koolschijn et al., 2009; Kempton et al., 2011; van der Werff et al., 2013). Amygdala alterations were also observed in studies reporting larger volumes related with early stress exposure, including with hemispheric differences (Pruessner et al., 2010; van der Werff et al., 2013). On the other hand, volume decreases in amygdala have also been associated with early and late-onset depressed subjects (Burke et al., 2011; van Uden et al., 2011). Importantly, we previously found in young subjects that stress triggers an atrophy of the caudate and the orbitofrontal cortex, but a hypertrophy of the putamen, changes that were shown to be reversible and accompanied by a functional reorganization after a stress-free period (Soares et al., 2012). Curiously, there is a very limited amount of information regarding the stress and mood-effects on structural aspects on the WM.

Herein we addressed the impact of normal aging, stress, mood and their interplay in the brain GM and WM structure.

## MATERIALS AND METHODS

### ETHICS STATEMENT

The present study was conducted in accordance with the principles expressed in the Declaration of Helsinki and was approved by the Ethics Committee of Hospital de Braga (Portugal). The study goals and tests were explained to all participants and all gave informed written consent.

### PARTICIPANTS, PSYCHOLOGICAL TESTS, AND CORTISOL MEASUREMENTS

This study assessed a sample of 104 participants [52 males and 52 females, mean age 65.20 ± 8.07, minimum age 51, and maximum 82, 5.43 ± 3.84 mean years of education and mean of 26.66 ± 3.30 Mini-Mental State Examination (MMSE; Folstein et al., 1975)] selected from a representative sample of the Portuguese population in terms of age, gender, and education, of the SWITCHBOX Consortium project (Santos et al., 2013). Participants responded to a laterality test and to a questionnaire regarding perceived stress (Perceived Stress Scale – PSS; mean 21.49 ± 8.18; Cohen et al., 1983). Participants were further assessed with the Geriatric Depression Scale (GDS, long version; mean 10.91 ± 6.70; Yesavage et al., 1982) by a certified psychologist.

### DATA ACQUISITION AND PROCESSING

Participants were scanned on a clinical approved Siemens Magnetom Avanto 1.5 T (Siemens Medical Solutions, Erlangen, Germany) at Hospital de Braga using the Siemens 12-channel receive-only head coil. The imaging session included one structural T1 high-resolution anatomical sequence, 3D MPRAGE (magnetization prepared rapid gradient echo). This protocol was performed with the following scan parameters: repetition time (TR) = 2.730 s, echo time (TE) = 3.48 ms, 176 sagittal slices with no gap, flip angle (FA) = 7°, in-plane resolution = 1.0 mm× 1.0 mm and slice thickness = 1.0 mm.

Before any data processing and analysis, all acquisitions were visually inspected by a certified neuroradiologist and confirmed that participants had no brain lesions and the acquisitions were not affected by critical head motion. Seven participants were excluded from the analysis based on the head motion and/or brain lesions.

Structural analysis based on segmentation of brain cortical and subcortical structures from T1 high-resolution anatomical data was performed using the Freesurfer toolkit version 5.1 (https://surfer.nmr.mgh.harvard.edu) running on an Ubuntu 12.04 LTS system. This software package implements a semi-automated segmentation workflow including processing stages such as spatial registration to the Talairach standard space, skull removal, normalization of WM intensity, tessellation of GM-WM segmentation, among others. For the cortical parcellation, two atlases are available: one gyral based atlas resulting in 68 structures (Desikan et al., 2006) and another considering the giral and sulcal parts as separate regions resulting in 148 different brain areas (Destrieux et al., 2010). For the present study the subcortical, WM, and gyral-based cortical segmentations were considered. The employed workflow has suffered several improvements in the past years (Fischl et al., 2002, 2004), is considered reliable across sessions, scanner platforms, updates and field strengths (Han and Fischl, 2007; Jovicich et al., 2009) and was already validated against manual segmentation procedures (Fischl et al., 2002).

### STATISTICAL ANALYSES

Statistical analyses [using the IBM SPSS Statistics software, v.22 (IBM, New York, NY, USA)] were performed with multiple regression models considering each volume as the dependent variable and age, gender, intracranial volume (ICV), PSS, GDS, and age^∗^PSS, age^∗^GDS and age^∗^PSS^∗^GDS interactions as independent variables. Additionally, in order to test any effect of the MMSE scores in the model, we included MMSE and the interaction MMSE^∗^GDS as independent variables. For each positive or negative correlation, the results were controlled for the other covariates. The key assumptions for multivariate linear regression analysis were met and the covariates were mean-centered to avoid multicollinearity issues (Aiken and West, 1991; Frazier et al., 2004).

Dissection of the two-way interactions was performed centring the PSS or GDS scores 1 SD below the mean, on the mean and 1 SD above the mean and assessing the age effect on brain volumetry in each model. In order to investigate the significant three-way interactions, the age effect was assessed in four different models: (1) centring both PSS and GDS scores one SD below the mean, (2) with the PSS scores centered one SD above the mean and GDS scores one SD below the mean, (3) PSS scores centered one SD below the mean and GDS one SD above the mean and (4) both variables centered one SD above the mean. Results were considered significant corrected for multiple comparisons using a False Discovery Rate (FDR) threshold of 0.05.

## RESULTS

### EFFECT OF AGE, STRESS, AND MOOD ON BRAIN VOLUMETRY

Volumetric analyses revealed that increased age was positively correlated with the volume of the choroid plexus (both sides), lateral ventricles and third ventricle and WM hypointensities. Most of the age correlations found were negative, including the total GM, cortical WM on both hemispheres, supratentorial volume, left accumbens, and both hippocampi (**Table 1**). The WM presented more alterations, but only negative correlations were observed with increasing age (**Table 2**). More specifically, significant volumetric decreases in the WM volume with increasing age were found in the orbitofrontal cortex, superior frontal, inferior and middle temporal, parahippocampal, posterior cingulate, and other frontal, parietal and temporal regions (**Table 2**).

**Table 1:** Effect of age on brain volumetry (corrected for multiple comparisons FDR 0.05).

Effect	Correlation	Region	*T*	*P*
Age	Positive	Choroid plexus (right)	5.8224	9.28E-08
		Lateral ventricle (left)	5,4376	4.78E-07
		White matter (WM) hypointensities	4.8414	5.47E-06
		Lateral ventricle (right)	4.6083	1.36E-05
		Choroid plexus (left)	3.7589	0.0003
		3^rd^ Ventricle	3.2458	0.0017
	Negative	Cortical WM (right)	-7.2174	1.78E-10
		Cortical WM	-6.9783	5.35E-10
		Cortical WM (left)	-6.5820	3.24E-09
		Supratentorial	-5.1779	1.41E-06
		Accumbens (left)	-4.6633	1.10E-05
		Hippocampus (left)	-4.0750	0.0001
		Hippocampus (right)	-3.8995	0.0001
		Total gray matter (GM)	-3.5415	0.0006


**Table 2:** Effect of age and stress on brain WM regional volumetry (corrected for multiple comparisons FDR 0.05).

Effect	Correlation	Region	*T*	*P*
Age	Negative	Lateral orbitofrontal (right)	-5.4714	4.15E-07
		Lateral orbitofrontal (left)	-5.7956	1.04E-07
		Superior frontal (left)	-5.6346	2.08E-07
		Inferior temporal (left)	-5.4901	3.83E-07
		Cerebellum (right)	-4.8172	6.02E-06
		Posterior cingulate (right)	-4.5550	1.68E-05
		Superior frontal (right)	-4.5026	2.05E-05
		Inferior temporal (right)	-4.2957	4.47E-05
		Paracentral (left)	-4.2858	4.65E-05
		Paracentral (right)	-4.2858	0.00090
		Medial orbitofrontal (right)	-4.2247	5.82E-05
		Parahippocampal (right)	-4.1981	6.57E-05
		Superior parietal (right)	-4.0156	0.00020
		Postcentral (left)	-3.9499	0.00015
		Entorhinal (left)	-3.9136	0.00018
		Middle temporal (right)	-3.9029	0.00001
		Superior parietal (left)	-3.8834	0.00020
		Pars triangularis (right)	-3.8736	0.00021
		Middle temporal (left)	-3.8476	0.00022
		Pars orbitalis (right)	-3.7961	0.00027
		Precentral (left)	-3.7331	0.00033
		Fusiform (left)	-3.7016	0.00037
		Postcentral (right)	-3.5270	0.00067
		Lateral occipital (right)	-3.4985	0.00074
		Cerebellum (left)	-3.4613	0.0008
		Inferior parietal (right)	-3.4307	0.00092
		Posterior cingulate (left)	-3.4172	0.00096
		Rostral middle frontal (left)	-3.3386	0.00012
		Rostral middle frontal (right)	-3.3753	0.00110
		Superior temporal (right)	-3.3266	0.00128
		Parahippocampal (left)	-3.2781	0.00150
		Pars opercularis (right)	-3.2039	0.00189
		Insula (right)	-3.0532	0.00300
		Lingual (right)	-3.0464	0.00305
		Pars triangularis (left)	-3.0312	0.00320
		Fusiform (right)	-2.9598	0.00037
		Frontal pole (right)	-2.9472	0.00410
		Lingual (left)	-2.9103	0.00457
		Supramarginal (left)	-2.8668	0.00525
		Supramarginal (right)	-2.8627	0.00525
		Inferior parietal (left)	-2.8547	0.0092
PSS	Negative	Frontal pole (right)	-3.3396	1.23E-03


The separate impact of stress (PSS score) and mood (GDS score) on brain volumetry did not reveal any significant changes, except for a decreased in WM volumetry in the right frontal pole with lower PSS scores (**Table 2**). A negative correlation between MMSE and GDS scores was found (*p* = 0.0002), however, none significant alteration was found when including MMSE and MMSE^∗^GDS in the model.

### INTERACTIONS OF AGE WITH STRESS AND MOOD ON BRAIN VOLUMETRY

**FIGURE 1:**
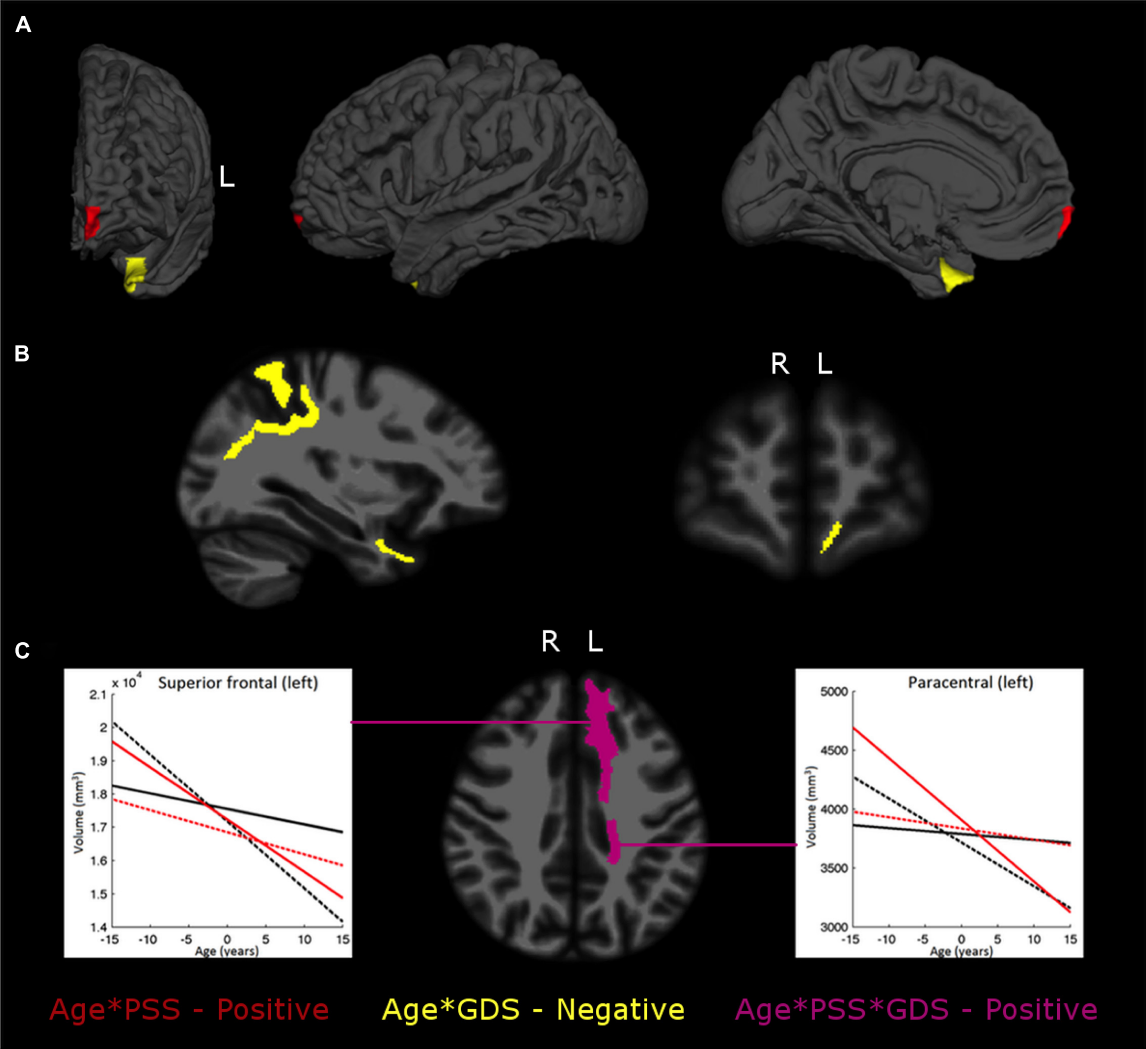
The interplay between stress or mood and aging in brain regional gray matter (GM) volumes **(A)**, white matter (WM) regions **(B),** and the Age*PSS*GDS interactions **(C)**. The images depict areas with significant interaction effects of age*PSS positive (in red), age*GDS negative (in yellow) and age*PSS*GDS positive (in violet). In **(C)** each graphic line is the regression line between age and regional volume for: Low PSS values (LPSS, black lines), combined with Low GDS (LPSS–LGDS, solid line), or with High GDS (LPSS–HGDS, dotted line); and High PSS value (HPSS, red lines), combined with Low GDS (HPSS–LGDS, solid line) or High GDS (HPSS–HGDS, dotted line).

Tests for the two-way interaction between age and PSS revealed a significant interaction with the volume of the left frontal pole (*p* = 0.0024). Further analysis revealed that for lower PSS scores, decreased volumes were observed with increasing aging; however, as PSS scores get higher the slope of increase with age also increases. In the left temporal pole (*p* = 0.0061), for low GDS scores, its volume increases with age, while for medium to high GDS scores there is a reduction that gets more pronounced as the scores get higher (**Table 3**; **Figure 1A**).

**Table 3:** Effect of the interplay between stress and aging and stress and mood on brain volumetry (corrected for multiple comparisons FDR 0.05).

Interaction	Correlation	Region	M–SD/M/M + SD	*T*	*P*
Age*PSS	Positive	Frontal pole (left)	-2.6821/3.5549/9.7919	3.1282	0.0024
Age*GDS	Negative	Temporal pole (left)	10.2711/-1.7243/-13.7198	-2.8121	0.0061


M, Mean; SD, standard deviation.

In the WM regional volumetry, only negative age^∗^GDS interactions were found (**Table 4**; **Figure 1B**). In the left frontal (*p* = 0.0018) and temporal pole (*p* = 0.0066), for low GDS scores, the WM volume increases with the age while for medium to high scores the volume decreases and the rate of decrease gets more prominent as the GDS scores get higher. In the left superior parietal (*p* = 0.0083) the WM volume decreases with age and this decrease gets more pronounced as GDS scores gethigher.

**Table 4:** Effect of the interplay between aging, stress, and mood on brain WM regional volumetry (corrected for multiple comparisons FDR 0.05).

Interaction	Correlation	Region	M–SD/M/M + SD	*T*	*P*
Age*GDS	Negative	Frontal pole (left)	1.1835/-0.9989/-3.1813	-3.2240	0.0018
		Temporal pole (left)	1.8836/-2.0358/-5.9551	-2.7813	0.0066
		Superior parietal (left)	-15.6389/-62.1391/-108.6391	-2.7026	0.0083
			LPSS_LGDS/LPSS_HGDS/HPSS_LGDS/HPSS_HGDS		
Age*PSS*GDS	Positive	Paracentral (left)	-5.0132/-37.1670/-52.2815/-9.5234	2.6634	0.0092
		Superior frontal (left)	-46.3864/-200.5990/-156.4589/-66.0731	2.6541	0.0093

*M, Mean; SD, standard deviation; LPSS, Low Perceived Stress Scale scores; HPSS, High Perceived Stress Scale scores; LGDS, Low Geriatric Depression Scale scores; HGDS, High Geriatric Depression Scale scores*.

The WM volume of the left paracentral (*p* = 0.0092) and the left superior frontal (*p* = 0.0093) regions showed three-way interactions, with both regions evidencing negative correlations between volume and age (**Table 4**; **Figure 1C**). Specifically, for low PSS scores, as GDS scores change from low to high the rate of volume decrease with age becomes more pronounced while for high PSS scores, the rate of volume decrease with age becomes less marked as GDS scores change from low to high.

## DISCUSSION

Several studies have consistently described the critical impact of the aging process, stress, and mood on brain volumetry. Nevertheless, most of the neuroimaging studies focused on the effect of individual elements, precluding the critical influence of the complex interplay among various processes. In this study, we dissected the influence of life events on brain GM and WM volumetry, namely stress (a more prolonged/chronic stress) and mood, throughout aging, and how they interplay and impact on brain structure.

With aging, we found a global, as well as a regional, pattern of volumetric GM and WM decrease, accompanied by an expansion of the ventricles, choroid plexus and CSF spaces, reflecting an atrophy of the brain parenchyma. Total and subcortical GM was decreased with age, including the left accumbens, both hippocampi and total cortical WM, frontal, temporal, occipital, and parietal WM regions also presented significant volumetric decreases with age. Similar findings have been consistently reported in the literature, both in cross-sectional and longitudinal studies (Raz et al., 2005; Smith et al., 2007; Abe et al., 2008; Fjell et al., 2009; Walhovd et al., 2011; Tamnes et al., 2013). Specifically, however, herein we found a higher decrease in global WM than GM, confirming a striker WM deterioration after the fifth decade (Gunning-Dixon et al., 2009; Lemaitre et al., 2012). This global WM volume decline, more pronounced in frontal regions (orbitofrontal, superior frontal, and rostral middle frontal) was paralleled by an increase with age of the WM hypointensities volume, a measure of lesion burden (Leritz et al., 2014).

It is well known that the stress impact is diverse on different life phases (Sousa and Almeida, 2012). Additionally, stress and mood are states known to be intrinsically connected and that interplay over the lifespan (Calabrese et al., 2009). In this study, and excluding any impact of the MMSE scores, in the volume of the left frontal pole GM, there was an inversion from decreases at low stress levels to increases at high levels with age. This result shows the critical stress impact in the frontal regions, especially at higher stress levels, leading to inversions from age-induced reductions to volume increases during aging (Cerqueira et al., 2007; Lupien et al., 2009). Importantly, behavioral stress affects, with possible reversibility, both structure and function of the prefrontal cortex, a region where neurons become less efficient with aging (McEwen and Morrison, 2013). On the other hand, on the left frontal pole WM, there is an increase in the volume reduction with age for higher depressive mood levels, in line with several previous findings (Konarski et al., 2008; Kong et al., 2014), showing the high susceptibility and variability of this region. Several studies have reported volumetric reductions in temporal regions associated with mood disorders (Drevets et al., 2008; Son et al., 2013) and herein we observed the depressive mood impact, especially at higher levels, in the increased volumetric reduction of the WM and GM in the left temporal pole. In this study we also found an increased volumetric reduction with age at high depressive mood levels in the left superior parietal WM, in line with the literature pointing to WM decreases in older patients with major depressive disorder (Zeng et al., 2012). Such lateralization effect, with more pronounced atrophy in structures localized in the left hemisphere, is in good line with previous studies on this topic (see for review, Cerqueira et al., 2008).

The most affected WM regions during aging by the interplay stress and mood are the left paracentral and the left superior frontal regions. Indeed, the superior frontal WM volume is known to present an accelerated decline with increasing age (Salat et al., 2009a). The volume of these regions decrease for all stress and mood level combinations, however, the decrease is much more pronounced for high stress and low mood levels. This indicates that for better mood (i.e., less depressive), the effect of stress is negative since it increases the negative relation between age and volume; this negative impact of stress does not seem to operate so obviously for subjects with higher depressive mood. These higher volumetric reductions with age, especially under high levels of stress, in WM of these regions, may be associated with the lower predisposition to action and extraception observed with increasing age. Importantly, the impact of stress and mood during the lifespan seems to be higher in the WM compared to the GM volumetry, in line with the described faster WM deterioration after the fifth decade of life (Gunning-Dixon et al., 2009).

This study presents also some important limitations. The analyses of stress and mood states were based only in psychological scales without any biological marker and indicator. Also, our sample included only middle aged to older adults and was a cross-sectional design, precluding a complete lifespan assessment, from childhood to elderly ages, and the evaluation of both individual differences and cohort effects. However, to the best of our knowledge there are no prior reports that evaluate the interplay between stress and mood on brain volumetry across the lifespan.

## CONCLUSION

In this study we have shown the critical influence of stress and mood, especially at higher levels, in brain volumetry. High levels of stress and/or mood may accelerate the typical age-induced decline or alternatively reduce the aging impact. We showed also that for the effects of stress and mood in brain volumetry, timing is crucial. Indeed, the clarification of the stress and mood interplay during aging may help to explain some divergent results associated with the aging decline. Moreover, we expect also to enlighten the association between abnormal volumetric alterations and several states that may lead to psychiatric disorders (Drevets et al., 2008; Konarski et al., 2008; Kempton et al., 2011; Kroes et al., 2011; Durkee et al., 2013; Kuhn and Gallinat, 2013)

## AUTHOR CONTRIBUTIONS

José M. Soares and Paulo Marques contributed in literature search, figures, study design, data collection, data analysis, data interpretation, and writing. Ricardo Magalhães contributed in data collection and data analysis. Nadine C. Santos and Nuno Sousa contributed in study design, data interpretation, and writing.

## Conflict of Interest Statement

The reviewer Josef Zihl declares that, despite having collaborated with co-authors Nadine C. Santos and Nuno Sousa, the review process was handled objectively. The authors declare that the research was conducted in the absence of any commercial or financial relationships that could be construed as a potential conflict of interest.
